# Alopecia and Iron Deficiency: An Interventional Pilot Study in Primary Care to Improve the Request of Ferritin

**DOI:** 10.1155/2020/7341018

**Published:** 2020-08-26

**Authors:** Maria Salinas, Maria Leiva-Salinas, Emilio Flores, Maite López-Garrigós, Carlos Leiva-Salinas

**Affiliations:** ^1^Clinical Laboratory, Hospital Universitario de San Juan, Carretera Nacional 322 s/n-03550-San Juan de Alicante, España, Spain; ^2^Department of Biochemistry and Molecular Pathology, Universidad Miguel Hernandez, Avda. Universidad, s/n. Edif. Torregaitán-2a Planta, 03202 Elche, Spain; ^3^Department of Dermatology, Hospital Marina Baixa, Av. Alcalde En Jaume Botella Mayor, 7, 03570 Villajoyosa, Spain; ^4^Department of Clinical Medicine, Universidad Miguel Hernandez, Carretera Nacional 322 s/n-03550-San Juan de Alicante, España, Spain; ^5^Department of Radiology and Medical Imaging, University of Missouri Health Care, Columbia, MO 65212, USA

## Abstract

**Background:**

The aim was to study the demographic and laboratory pattern of primary care patients with alopecia undergoing laboratory testing, more specifically, the request of hemoglobin and ferritin and values showing anemia and iron deficiency, and to evaluate the effects of an intervention involving automatic ferritin registration and measurement when not requested.

**Methods:**

Retrospective and prospective observational cross-sectional studies were conducted, as well as an intervention to automatically register and measure ferritin when not requested by the general practitioner.

**Results:**

There were 343 and 1032 primary care laboratory requests prompted by alopecia in the retrospective and prospective studies. Hemoglobin was requested in almost every patient and ferritin in 88%. 5% of the cohort had anemia, and 25% had iron deficiency. The intervention registered and measured that 123 ferritin and 24 iron deficiencies were detected in patients with alopecia, all women, at a cost of 10.6€.

**Conclusion:**

Primary care patients with alopecia and laboratory tests request were mainly young female. Our intervention added ferritin when not requested, detecting iron deficiency in 27.9% of women, potentially avoiding the adverse effects of iron deficiency on hair loss.

## 1. Introduction

Hair loss is a common health problem and represents a diverse group of disorders with different etiologies, as a result of a variety of local and systemic conditions [1]. The first step in the work-up of alopecia is an early diagnosis through a detailed clinical history and physical examination and appropriate laboratory testing [2, 3], to identify the cause. This allows for timely, adequate, and effective treatment and the decision/need for long-term management/follow-up [4]. Indeed, an optimal hair growth potential exists when specific parameters for biochemical variables are operating [4].

Although there is insufficient evidence to recommend universal screening for iron deficiency in patients with hair loss, iron deficiency is a frequent cause of alopecia [5].

According to the World Health Organization (WHO), in the Spanish population, anemia prevalence ranges from 14% to 18% in children and in women of reproductive age, respectively [6]. Román-Viñas et al. observed a prevalence of iron intake inadequacy of 10%–21% of the estimated average requirement (EAR) in a number of European populations when analyzing the European Nutrition and Health Report [7].

Although serum ferritin is not only the primary diagnostic parameter for microcytic anemia but also for iron deficiency [4, 8], it frequently remains undiagnosed when occurs without anemia, as symptoms are often only attributed to the iron deficiency accompanied with anemia [9]. In fact, values below 10 mg/l represent depleted iron stores and between 10 and 30 mg/l can confirm deficiency [9]. Low-serum ferritin is associated with alopecia in women [10] as it might be in men [5].

New developments in information technologies such as patient electronic medical history (PEMH), laboratory information system (LIS) [11], and computerized physician order entry (CPOE) [12] could be used to improve test laboratory request, and consequently, the diagnosis, and hence patient outcome, as the number of laboratory requests with patient clinical information has greatly increased [13]. Consequently, it is now possible to automatically customize the requests according to the patient suspected diagnosis or current situation [14–16].

Our aims were, first, to study the demographic profile of primary care patients with alopecia who underwent laboratory blood testing and investigate their hemoglobin and ferritin request and values showing anemia and iron deficiency, and second, to improve the laboratory contribution to alopecia through the automatic ferritin registration and measurement when not requested by the general practitioners (GPs).

## 2. Materials and Methods

### 2.1. Study Design

Retrospective and prospective observational cross-sectional studies were conducted from July 2016 to June 2017 and from July 2017 to March 2020, respectively. An intervention was also undertaken in the last period (July 2017–March 2020).

### 2.2. Laboratory and Hospital Characteristics

The laboratory is located at the public University Hospital of San Juan (Alicante, Spain), a 370-bed suburban community hospital that serves a population of 234551 inhabitants, including nine different primary care centers (PCCs). It receives samples from inpatients, outpatients, and primary care patients. Primary care samples are transported by couriers from the different PCCs to the laboratory reception desk.

Laboratory requests are made through CPOE that offers the GPs a field regarding the reason for the laboratory request through International Classification of Diseases, Ninth Revision, Clinical Modification (CIE-9-MC) codes [17]. The laboratory reports are automatically sent to the PEMH.

### 2.3. Participants

All community inhabitants were covered by the clinical laboratory.

### 2.4. Retrospective and Prospective Studies

In both periods, we assessed the number of primary care patients with a laboratory request due to alopecia (ICD-9: 783.0 [18]), their demographic data, the number of hemoglobin and ferritin requested, and values showing anemia and iron deficiency (hemoglobin values below 12 for female and 13.5 g/dL for male; ferritin values below 30 ng/mL) through a LIS search.

### 2.5. Intervention Design

**Figure 1:**
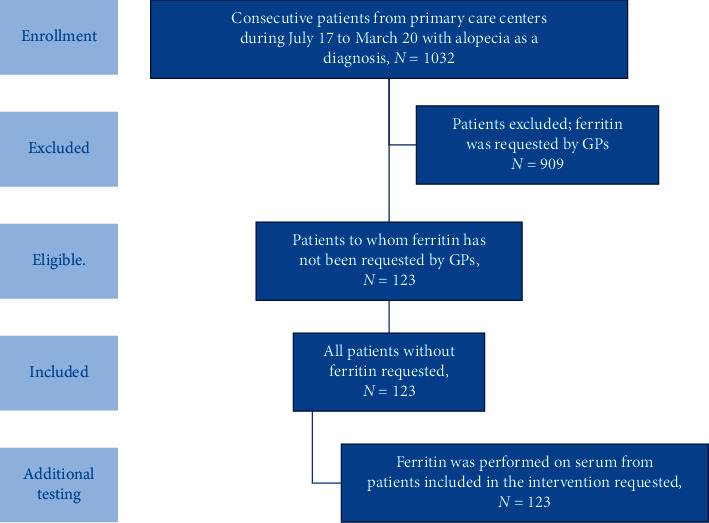
Flow chart of intervention and study design.

An intervention was designed according to the retrospective study results, in agreement with GPs. The LIS would automatically register ferritin when not ordered by GPs, to all requests from primary care patients with alopecia as a diagnosis (ICD-9: 783.0) and without ferritin measurement in the previous year (Figure 1).

### 2.6. Data Analysis

Demographic data, the number of hemoglobin and ferritin requests, and values were assessed and compared between the retrospective and prospective study; we also evaluated the number of patients with anemia and iron deficiency.

In the intervention, we counted the additionally registered ferritin and the number of total identified patients with iron deficiency. We calculated the total economic cost per identified patient, taking into account the total number of additional serum ferritin tests that were measured (*N*) and reagent cost (2.04€ per ferritin test) (cost per identified patient = *N* *∗* 2.04€/number of identified patients).

The study was approved by the Hospital Research Committee.

### 2.7. Outcome measures

We evaluated the demographic data, number of requests for hemoglobin and ferritin, and values showing anemia or iron deficiency. We also counted the additionally registered ferritin and the number of total identified patients with iron deficiency through the intervention.

### 2.8. Laboratory Methods

Three milliliters (3 ml) of blood sample were collected from each of the subjects into BD Vacutainer® K_2_EDTA tubes (Becton, Dickinson and Company, Franklin Lakes, NJ, USA) to analyze the CBC on Sysmex XE 2100 analyzer (Sysmex, Kobe, Japan). The serum concentration of ferritin was measured on BD Vacutainer® Serum Separating Tubes II Advance Tube (SST) (Becton, Dickinson and Company, Franklin Lakes, NJ, USA), using an immunoassay on Modular E170 (Roche Diagnostics, Indianapolis, IN).

### 2.9. Statistical Methods

The statistical analyses were conducted with SPSS version 20 for windows (SPSS Inc, Chicago, IL). The statistical analysis included a descriptive analysis of the variables. Normal quantitative variables were expressed as average and standard deviation. Categorical variables were expressed as percentages.

## 3. Results

### 3.1. Retrospective Study

There were 343 primary care laboratory requests prompted by alopecia. Patients were mainly young female; however, the male cohort was significantly younger (Table 1).

**Figure 2:**
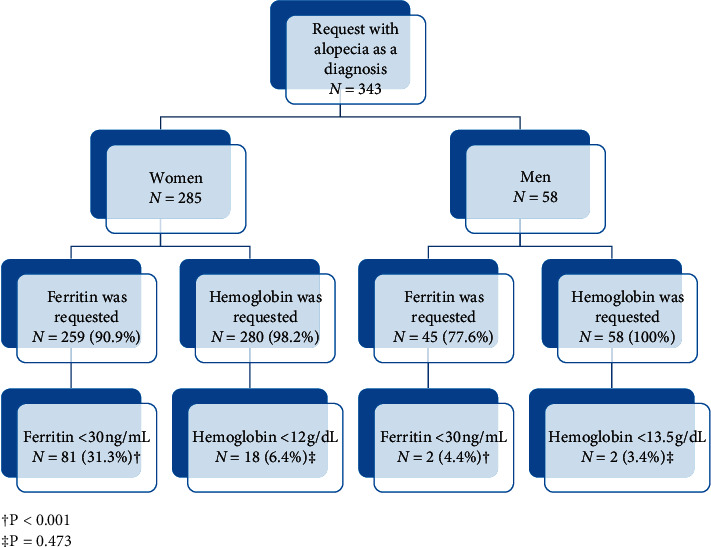
Results of the retrospective study legend: ^†^*P* < 0.001 and ^‡^*P*=0.473.

Hemoglobin was requested to 338 (98%) patients and ferritin to 304 (88%). Twenty (5.8%) and 83 (24.2%) patients showed anemia and iron deficiency, respectively. Ferritin was more frequently requested in women. The percentage of woman with anemia and iron deficiency was higher (Figure 2). Median values of hemoglobin and ferritin are shown in Table 1.

### 3.2. Prospective Study

One thousand thirty-two laboratory requests were received from primary care due to alopecia. Patient's demographic characteristics were very similar to that observed in the retrospective study (Table 1). Table 1 also shows the hemoglobin and ferritin values that did not differ from those found in the retrospective study.

Hemoglobin was requested to 1023 (99.1%) patients and ferritin to 909 (87.2%). Fifty-nine (5.8%) and 235 (25.9%) patients showed anemia and iron deficiency, respectively; percentage that did not differ between the prospective and the retrospective study.

**Figure 3:**
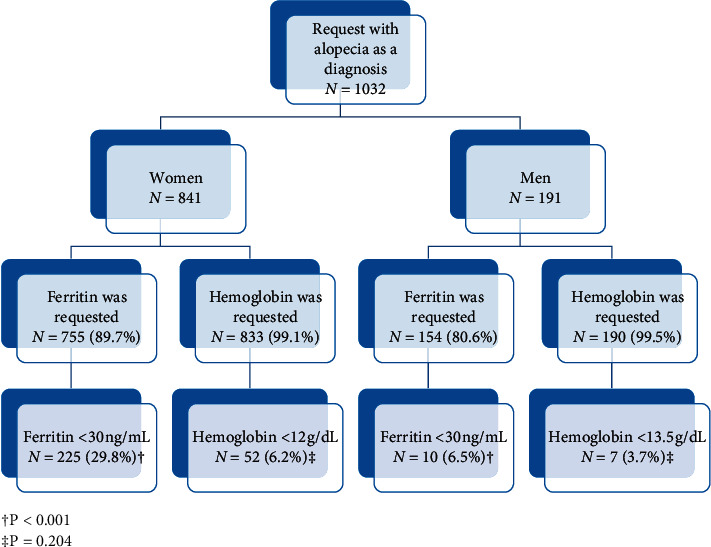
Results of the prospective study legend: ^†^*P* < 0.001 and ^‡^*P*=0.204.

As shown in the retrospective research, ferritin was more frequently requested in women, and the percentage of women with anemia and iron deficit was higher (Figure 3).

### 3.3. Intervention Results

Figure 1 shows a flow chart of intervention design. One hundred twenty-three (11.9%) ferritin tests were automatically registered though our intervention (Table 2) to 86 female and 37 male patients. Median ferritin value was 50 ng/mL and 167 ng/mL for women and men, respectively. In total, through our intervention, we detected iron deficiency in 24 (19.5%) of cases of ferritin that were automatically registered. All were women with a 27.9% showing iron deficiency. Each detected case of iron deficiency resulted in cost of 10.6€.

## 4. Discussion

This is the first effort to study primary care patients with alopecia undergoing laboratory testing, the number of hemoglobin and ferritin requests, and values of anemia and iron deficiency. The majority were female and young. A possible reason for this is that women are more likely to consult for hair diseases due to the aesthetic implications they involve. Hemoglobin was requested in 99% and ferritin in 80% of subjects. Despite the small prevalence of anemia, there were a significant number of subjects with iron deficiency based on ferritin values, especially women. The computerized aided intervention through the automatic addition of ferritin identified around one-fifth of patients with iron deficiency, all female.

The fact that most primary care patients with alopecia were women is possibly related to their lifetime history of iron loss due to heavy menstrual bleeding or multiple pregnancies [19], as 10–20% of menstruating women have iron deficiency, and 3–5% of them are frankly anemic [20]. However, regarding the observed demographic pattern in men—young adults—more studies are needed to provide a rationale, as the majority of evidence regarding alopecia involves women [10, 21]. In Spain, the observed levels of total median daily iron intake were low for women and also for men [22].

Our study showed that even if a small number of the patients had anemia, a higher proportion presented iron deficiency. The population of the study was from Valencian community, one of the most important autonomous communities by volume of GDP, occupying the 4th position in the GDP ranking of the autonomous communities [23] with a medium level of adherence to Mediterranean diet [24]. Iron deficiency anemia is a late manifestation of iron deficiency, both of which are common medical conditions in everyday clinical practice [20, 25]. This result corroborates that the request of serum ferritin concentration—the most sensitive and specific test used for the identification of iron deficiency [25, 26]—is needed independently of the presence of anemia.

There is an overall over-request of anemia chemistry tests in primary care in Spain [27] as more than one ferritin is ordered for every three blood counts [28]. However, as observed in young primary care patients with anorexia [29], our results show that despite the observed overall over request, there are primary care patients with alopecia who do not undergo a key test such as ferritin to evaluate for iron deficiency. Some studies suggest that iron deficiency may be related to alopecia and diffuse hair loss [10, 30–32], whereas others do not [33–35]. Although there is no current evidence to recommend universal screening for iron deficiency in patients with hair loss [5], our study results and the high percentage of women with iron deficiency suggest the need to measure ferritin in women consulting GPs because of alopecia. The study had certain limitations. First, we could not analyze the relationship between iron deficiency and different types of alopecia. C-reactive protein and erythrocyte sedimentation rate were not evaluated in all subjects; consequently, selection bias is a potential significant confounder. Second, if no laboratory testing is ordered during the patient encounter, the opportunity to assess ferritin and other laboratory tests may be lost. Finally, the calculated economic costs of the study may not apply to other settings, since our laboratory belongs to a public health network, in which reagent prices are relatively low.

The majority of primary care patients with alopecia undergoing laboratory testing were female and young. Hemoglobin was requested in nearly every subject and ferritin in 80%, with a small prevalence of anemia but a significant number of cases of iron deficiency based on ferritin values, especially in women. The LIS successfully played a role in the automatic registration of ferritin in patients with alopecia when the referring physician did not do so, detecting iron deficiency in a quarter of women, potentially avoiding the adverse effects of iron deficiency.

## Figures and Tables

**Table 1:** Demographic characteristics of patients in the retrospective and prospective study and ferritin and hemoglobin median values.

	Retrospective study			Prospective study		
	Women	Men	*P* value	Women	Men	*P* value
*N*; (%)	285; 83.1%	58; 16.9%		841; 81.5%	191; 18.5%	
Age (median; IQR)	39; 26	26; 19	<0.001	41; 29	26; 17	<0.001
Ferritin ng/mL (median; IQR)	45; 56	133; 84.5		52; 67	147; 118	
Hemoglobin g/dL (median; IQR)	13.5; 1.1	15.7; 1.3		13.5; 1.3	15.3; 1.4	

**Table 2:** Number of ferritin added through intervention, median value of ferritin added, and percentage of ferritin <30 ng/mL.

	Women	Men	*P* value
Ferritin added through intervention *N*; (%) of patients in the prospective study	86; 10.2%	37; 19.4%	
Ferritin ng/mL (median; IQR)	50; 101	167; 142	
Ferritin <30 ng/mL (*N*; (%))	24; 27.9%	0; 0%	<0.001

## Abbreviations

LIMS: Laboratory information management system
PEMR: Patient electronic medical history
CPOE: Computerized physician order entry
GPs: General practitioners
PCCs: Primary care centers
ICD: International Classification of Diseases.

## References

[1] Bermudez-García L., Justel-Pérez J. P., Pérez-Mansilla I. (2011). *Guía Clínica de Alopecia*.

[2] Ahanogbe I., Gavino A. C. P. (2015). Evaluation and management of the hair loss patient in the primary care setting. *Prim Care*.

[3] Nielson T. A., Reichel M. (1995). Alopecia: diagnosis and management. *Am Fam Physician*.

[4] Rushton D. H. (1993). Management of hair loss in women. *Clinics in Dermatology*.

[5] Trost L. B., Bergfeld W. F., Calogeras E. (2006). The diagnosis and treatment of iron deficiency and its potential relationship to hair loss. *Journal of the American Academy of Dermatology*.

[6] De Benoist B., Mclean E. (2008). *Worldwide Prevalence of Anaemia 1993-2005 Who Global Database on Anaemia*.

[7] Roman Viñas B., Ribas Barba L., Ngo J. (2011). Projected prevalence of inadequate nutrient intakes in Europe. *Annals of Nutrition and Metabolism*.

[8] AEFA (1995). *Guidelines for the Use of Serum Tests of Iron Stores*.

[9] Fehr J., Favrat B., Schleiffenbaum B., Krayenbühl P. A., Kapanci C., Von Orelli F. (2009). Diagnosis and treatment of iron deficiency without anaemia. *Revue Médicale Suisse*.

[10] Kantor J., Kessler L. J., Brooks D. G., Cotsarelis G. (2003). Decreased serum ferritin is associated with alopecia in women. *Journal of Investigative Dermatology*.

[11] Salinas M., López-Garrigós M., Asencio A., Leiva-Salinas M., Lugo J., Leiva-Salinas C. (2015). Laboratory utilization improvement through a computer-aided algorithm developed with general practitioners. *Clinical Chemistry and Laboratory Medicine*.

[12] Rodriguez-Borja E., Villalba-Martinez C., Barba-Serrano E., Carratala-Calvo A. (2016). Failure to review STAT clinical laboratory requests and its economical impact. *Biochemia Medica*.

[13] Salinas M., López-Garrigós M., Flores E. (2016). Indications for laboratory tests in primary care: assessment of the most frequent indications and requests with blank clinical information. *Biochemia Medica*.

[14] Salinas M., López-Garrigós M., Flores E., Ahumada M., Leiva-Salinas C. (2019). Laboratory intervention to improve the request of urinary albumin in primary care patients with arterial hypertension and financial implications. *Clinical Biochemistry*.

[15] Salinas M., López-Garrigós M., Flores E., Lugo J., Leiva-Salinas C. (2019). Laboratory computer-based interventions for better adherence to guidelines in the diagnosis and monitoring of type 2 diabetes. *Diabetes Therapy*.

[16] Salinas M., López-Garrigós M., Flores E., Blasco A., Leiva-Salinas C. (2020). Less is more: two automated interventions to increase vitamin B12 measurement when long-term proton pump inhibitor and decrease redundant testing. *Clinica Chimica Acta*.

[17] National Center for Health Statistics (2013). *Icd-ICD-9-CM-International Classification of Diseases, Ninth revision, Clinical Modification*.

[18] Instituto de Información Sanitaria (2004). *Boletines de Codicación Clínica con la CIE-9-MC*.

[19] Hershko C., Camaschella C. (2014). How I treat unexplained refractory iron deficiency anemia. *Blood*.

[20] Umbreit J. (2005). Iron deficiency: a concise review. *American Journal of Hematology*.

[21] Deloche C., Bastien P., Chadoutaud S. (2007). Low iron stores: a risk factor for excessive hair loss in non-menopausal women. *European Journal of Dermatology*.

[22] Samaniego-Vaesken M. d. L., Partearroyo T., Olza J. (2017). Iron intake and dietary sources in the Spanish population: findings from the ANIBES study. *Nutrients*.

[23] Instituto Nacional de Estadística, http://www.ine.es

[24] Bernat N. S. O., Trescastro-López E. M., Izquierdo J. Q. (2019). Different classification of an adult population by two validated indexes of adherence to the mediterranean diet. *Nutricion Hospitalaria*.

[25] Camaschella C. (2019). Iron deficiency. *Blood*.

[26] Lopez A., Cacoub P., Macdougall I. C., Peyrin-Biroulet L. (2016). Iron deficiency anaemia. *Lancet*.

[27] Salinas M., López-Garrigós M., Flores E., Leiva-Salinas C. (2017). Primary care requests for anaemia chemistry tests in Spain: potential iron, transferrin and folate over-requesting. *Journal of Clinical Pathology*.

[28] Salinas M., López-Garrigós M., Flores E., Uris J., Leiva-Salinas C. (2015). Potential over request in anemia laboratory tests in primary care in Spain. *Hematology*.

[29] Salinas M., López-Garrigós M., Flores E., Leiva-Salinas C. (2019). Automated requests for thyroid-stimulating hormone and ferretin tests in young primary care patients with anorexia as an intervention to improve detection of underlying conditions. *Laboratory Medicine*.

[30] Rushton D. H. (2002). Nutritional factors and hair loss. *Clinical and Experimental Dermatology*.

[31] Rushton D. H., Norris M. J., Dover R., Busuttil N. (2002). Causes of hair loss and the developments in hair rejuvenation. *International Journal of Cosmetic Science*.

[32] White M. I., Currie J., Williams M. P. (1994). A study of the tissue iron status of patients with alopecia areata. *British Journal of Dermatology*.

[33] Aydingbz I. E., Ferhanoğlu B., Güney O. (1999). Does tissue iron status have a role in female alopecia?. *Journal of the European Academy of Dermatology and Venereology*.

[34] Boffa M. J., Wood P., Griffiths C. E. M. (2006). Iron status of patients with alopecia areata. *British Journal of Dermatology*.

[35] Bregy A., Trüeb R. M. (2008). No association between serum ferritin levels >10 *μ*g/l and hair loss activity in women. *Dermatology*.

